# Modeling and Comparative Analysis of Atmospheric Pressure Anodic Carbon Arc Discharge in Argon and Helium–Producing Carbon Nanostructures

**DOI:** 10.3390/nano13131966

**Published:** 2023-06-28

**Authors:** Almaz Saifutdinov, Boris Timerkaev

**Affiliations:** Department of General Physics, Kazan National Research Technical University named after A.N. Tupolev-KAI, K. Marx St., 10, 420071 Kazan, Russia

**Keywords:** arc discharge, carbon, unified model, extended fluid model

## Abstract

In this work, within the framework of a unified model for the discharge gap and electrodes, a comparative numerical analysis was carried out on the effect of evaporation of graphite anode material on the characteristics of the arc discharge in helium and argon. The effect of changing the plasma-forming ion, in which the ion of evaporated atomic carbon becomes the dominant ion, is demonstrated. For an arc discharge in helium, this effect is accompanied by a jump-like change in the dependence of the current density on voltage (CVC), and smoothly for a discharge in argon. With regard to the dynamics of the ignition of an arc discharge, it is shown that during the transition from glow discharge to arc in helium, the discharge parameters are also accompanied by an abrupt change, while in argon, this transition is smooth. This is due to the fact that the ionization potentials, as well as the ionization cross sections, differ significantly for helium and carbon, and are close in value for helium and argon. For various points on the CVC, the density distributions of the charged and neutral particles of an inert gas and evaporated gases are presented.

## 1. Introduction

Nanotechnologies are a rapidly developing area of modern science. The goal of modern nanotechnologies is a comprehensive study of the processes and products of the synthesis of nanostructures and nanostructured materials with controlled functional properties [1,2,3,4,5,6,7,8,9,10,11,12,13]. Plasma synthesis of nanostructures has great potential compared to traditional (CVD, high temperature and high pressure, liquid phase, etc.) methods of obtaining nanoparticles, since it provides high throughput, short nanostructure growth time, optimized material properties, and low cost (especially in case of synthesis in a gas discharge plasma at atmospheric pressure). These features of plasma synthesis are due to the possibility of supporting the production of nanostructures with a higher yield both in the plasma volume and at its boundaries (walls limiting the plasma volume, open discharge boundaries, “plasma-gas”), and also often at high parameter gradients (temperature, concentrations, electric fields) of the working medium and higher chemical purity, compared, for example, with CVD [3]. Moreover, the production of nanostructures using non-equilibrium plasma, in which plasma particles, including ions, electrons, excited and neutral particles, and radicals, are at different temperatures, is especially attractive, since it provides non-thermal synthesis of a wide range of nanomaterials, both with high and low melting temperatures [1,2,3,6,7,8,10,11,12,13], and at low and high pressures.

At present, various types of discharges are used in the problems of plasma synthesis of nanostructures and nanomaterials [10,11,12,13]. At the same time, an arc discharge at atmospheric pressure in inert gases stands out among other methods for the synthesis of nanostructures due to the high productivity, wide variety and high quality of the obtained nanomaterials, mainly core–shell nanoparticles, monolayers of transition metal dichalcogenides, and carbon nanostructures such as graphene, carbon nanotubes, and nanodiamonds [14,15,16,17,18]. The unique capabilities of the electric arc method are due to its flexibility and a wide range of plasma parameters. In particular, starting with the pioneering work of Iijima on the synthesis of carbon nanotubes using an atmospheric pressure arc discharge [19], the production of nanomaterials such as boron nitride nanotubes (BNNT) [20] and molybdenum disulfide nanoparticles [21] has contributed to the development of plasma nanotechnologies.

At present, plasma nanotechnologies have acquired an interdisciplinary character, and are used in the process of creating nanostructures and nanomaterials for optoelectronic applications [22,23,24,25], smart materials [26,27], medicine [28,29], and the modeling of nanomaterials [30]. On the other hand, the problem associated with the complete control of the quality and reproducibility of the synthesis of low-dimensional materials remains unsolved. Fundamental questions related to the optimal values of plasma parameters under the conditions of nanoparticle synthesis, plasma diagnostics, etc., also remain unresolved. On the other hand, the development of the existing self-consistent physical and mathematical models of the arc discharge and the performance of full-scale numerical experiments have already played a decisive role in understanding the effect of experimental parameters on the kinetics of nanoparticle growth [31]. To date, there are various models of arc discharges in 0D [32], 1D and 2D formulations in the framework of LTE and non-LTE approximations. For quite a long time, it was the LTE approximation that was used in modeling arc discharges, which is still used in scientific research [33,34,35,36,37]. On the other hand, a series of experimental and theoretical works showed a deviation from the local thermodynamic equilibrium [38,39,40] at the periphery of the arc discharge and in the near-electrode regions. In this regard, completely nonequilibrium models of the arc discharge that take into account near-electrode effects are being developed [41,42,43,44,45,46,47].

Fully nonequilibrium models are correct not only for the arc column in its central part, but also for the plasma periphery and near-electrode regions [41,42]. Regions with a predominance of space charge (near-cathode and near-anode layers) are considered using local (0D) models, self-consistently related to arc modeling, on the one hand, and to modeling electrodes with an arc [43,44,45,46]. A review of studies on nonequilibrium arc plasma and a comparison of two-temperature models and completely nonequilibrium models are given in [47].

At present, self-consistent models of arc discharges have been formulated, which describe, in a unified way, the processes occurring in the discharge gap and in electrodes and take into account conjugated effects [48,49,50,51,52,53,54,55,56,57]. Such models are presented both in one-dimensional and two-dimensional formulations. Arc discharges with a contracted and diffuse current spot were reproduced [48]. To develop these models, it is necessary to take into account the ablation of electrodes (in particular, the anode) and the deposition on the surface (cathode) of the evaporated material.

The construction of such a model assumes both fundamental and applied interest. In particular, such models from one side will help to fully describe the non-equilibrium processes occurring in an arc discharge, which are often ignored in the framework of LTE approximations. On the other hand, this model will serve as a tool for predicting plasma parameters under the conditions of synthesis of carbon nanostructures, as well as for optimizing modern compact plasma-chemical reactors for the synthesis of carbon nanostructures [17].

Therefore, the aim of the presented work is to formulate a self-consistent physical and mathematical model of an arc discharge in inert gases with graphite electrodes, which takes into account the ablation of the electrodes and the evaporation of the electrode material into the discharge gap. It aims to carry out a comparative analysis of the parameters of an arc discharge in inert gases (argon and helium), taking into account the evaporation of carbon particles into the discharge gap.

## 2. Model Description

### 2.1. Model Equations and Boundary Conditions

To determine the distributions of the spatial characteristics of the DC discharge, a self-consistent model based on the extended fluid description of plasma was formulated, which is unified from the point of view of describing the discharge gap and electrodes. It includes k densities’ balance equations for all types of considered particles (neutral, excited particles, electrons and ions), $n_{k}$ of the buffer gas (with index “buf”), as well as gas from particles evaporated from the surface of the electrodes (with index “C”), the balance equation electron energy density $n_{\varepsilon }$, and Poisson’s equation for the electric potential *φ*. To describe gas heating, two equations for the energy balance of heavy plasma particles are formulated: for helium or argon, and for particles of evaporated gas. In addition, the model includes heat conduction equations for the cathode and anode. Thus, the system of equations takes the following form:

(1) $$\frac{\partial n_{k}}{\partial t}+\nabla \cdot \Gamma_{k}=\sum_{j=1}^{N_{r}}\left(a_{kj}^{R}-a_{kj}^{L}\right)k_{j}\prod_{k=1}^{N_{s}}n_{k}^{\nu_{kj}^{L}},$$

(2) $$\frac{\partial n_{\varepsilon }}{\partial t}+\nabla \cdot Q_{\varepsilon }=-eE\cdot \Gamma_{e}-Q_{el,e-\mathrm{buf}}-Q_{el,e-C}-Q_{in},$$

(3) $$\frac{\partial }{\partial t}\left(\sum_{k\neq e,C}n_{k}C_{vk}T_{\mathrm{buf}}\right)+\nabla \cdot q_{\mathrm{buf}}=\sum_{k\neq e,C}ez_{k}\Gamma_{k}\cdot E+Q_{el,e-\mathrm{buf}}-Q_{el,\mathrm{buf}-C},$$

(4) $$\frac{\partial }{\partial t}\left(\sum_{k\neq e,\mathrm{buf}}n_{k}C_{vk}T_{C}\right)+\nabla \cdot q_{C}=\sum_{k\neq e,\mathrm{buf}}ez_{k}\Gamma_{k}\cdot E+Q_{el,e-C}+Q_{el,\mathrm{buf}-C}+Q_{chem}+Q_{rec},$$

(5) $$\Delta \varphi =-\frac{e}{\varepsilon_{0}}\left(\sum_{k=1}^{N}z_{k}n_{k}-n_{e}\right),\;E=-\nabla \varphi ,$$

(6) $$\rho_{c,a}c_{p\;c,a}\frac{\partial T_{c,a}}{\partial t}-\nabla \cdot \left(\Lambda_{c,a}\nabla T_{c,a}\right)=Q_{c,a}.$$

Here, the right side of Equation (1) describes the change in the number of particles of type *k* due to the reaction *j*, where $a_{kj}^{L}$ and $a_{kj}^{R}$ are stoichiometric coefficients; it is determined through the reaction constant. The summation is carried out over all reactions *j* occurring in the discharge, and the product is over all types of particles participating in the reaction. E is the electric field strength, the distribution of which is determined from the connection with the potential determined from the Poisson Equation (5), *e* is the charge of the electron, and ε_0_ is the dielectric constant; *z_k_* is the dimensionless charge number of a particle of type *k*. The electron energy density is defined as $n_{\varepsilon }=n_{e}\bar{\varepsilon }$, where $n_{e}$ is the density of electrons, $\bar{\varepsilon }$ is the average energy of the entire ensemble of electrons $n_{e}$. The electron temperature *T_e_* = 2/3$\bar{\varepsilon }$ is understood as 2/3 of the average energy of the entire ensemble. The density fluxes of charged, excited, and neutral particles $\Gamma_{k}$ in Equation (1), where *k* = *e*, *i*, *n*, as well as the electron energy density flux $Q_{\varepsilon }$ in Equation (2), respectively, are written in the diffusion–drift approximation

(7) $$\Gamma_{k}=-D_{k}\nabla n_{k}+z_{k}\mu_{k}E_{s}n_{k},$$

(8) $$\Gamma_{n}=-D_{n}\nabla n_{n}\;,$$

(9) $$Q_{\varepsilon }=-D_{\varepsilon }\nabla n_{\varepsilon }-\mu_{\varepsilon }En_{\varepsilon }\;,$$

where $D_{e},D_{i}$ are the diffusion coefficients of electrons and ions, $D_{n}$ are the diffusion coefficients of excited and neutral plasma particles, $\mu_{e},\mu_{i}$ are the mobility of charged particles in an electric field, $\mu_{\varepsilon }$ is the electron energy mobility, and $D_{\varepsilon }$ is the electron energy diffusion coefficient.

The first terms on the right side of (2)–(4) describe the Joule heating of electrons, heavy buffer gas particles and heavy carbon gas particles, respectively. The terms $Q_{el,e-\mathrm{buf}}$ and $Q_{el,e-C}$ in (2) describe the energy exchange during elastic collisions of electrons with neutral gas particles. The last term on the right side of (2) $Q_{in}=\sum_{j}\Delta \varepsilon_{j}R_{j}$ describes the change in energy due to inelastic collisions of electrons and heavy plasma particles, and is defined as an inelastic process involving an electron *R_j_* = *k_j_*(*T_e_*)*n_e_n_n_*, where *n_n_* is the kind of neutral particle.

The fluxes included in the energy balance equations for the heavy plasma component in (3) and (4) were written in the following form:

(10) $$q=-\sum_{k\neq e,C}\Lambda_{k}\nabla T+\sum_{k\neq e,C}C_{pk}T\;\Gamma_{k},$$

(11) $$q_{C}=-\Lambda_{C}\nabla T_{vap}+\sum_{k\neq e,\mathrm{buf}}C_{pk}T_{C}\;\Gamma_{k}.$$

Here, Λ and $\Lambda_{C}$ are the thermal conductivities of the buffer gas and the gas of particles of the evaporated material (carbon), whose values were determined as functions of temperature based on the data in [58]. The second terms on the right-hand sides in Equations (10) and (11) correspond to the energy density fluxes due to the diffusion of molecules. $C_{pk}$ is the heat capacity of a gas (buffer or carbon) at constant pressure.

For carbon particles evaporated from graphite electrodes, the terms $Q_{chem}$ and $Q_{rec}$ were additionally taken into account. The term in (4) $Q_{chem}$ describes the energy lost or gained by carbon gas as a result of exothermic and endothermic chemical reactions, and $Q_{rec}$ refers to the energy gained as a result of dissociative recombination reactions.

In Equation (6), the term $Q_{c,a}$ on the right side is the source of heating of the electrodes due to resistive losses, which is calculated from the continuity equation for the current density:

(12) ∇⋅J=0

where $J=\sigma_{c,a}E_{c,a}$ is the current density, and $\sigma_{c,a}$ is the conductivity of the metal electrode. The electric field is expressed in terms of the potential of the electric field in the electrode $E_{c,a}=-\nabla \varphi_{c,a}.$ Thus, $Q_{c,a}=J_{c,a}\cdot E_{c,a}$.

In numerical calculations, a one-dimensional computational domain was considered; this is similar to that considered by us in our previous work [49]. The boundary conditions for Equations (1)–(6) were written in a similar manner to [49]. However, when the electrode surface reaches the melting temperature, a phase transition and evaporation of the electrode material into the gas discharge gap begins. In this regard, it is necessary to take into account additional factors. Thus, for Equation (6), the boundary condition on the cathode surface from the side of the plasma region (x = 0) was written as follows

(13) $$n\cdot Q_{c}=n\cdot \left(\sum_{i}^{}Q_{i}+Q_{evp}+Q_{dep}\right)$$

Here, the first term on the right-hand side is described in detail in [48,49]. It includes the energy flux density transferred by ions to the cathode, the heat flux density transferred by the heated gas (plasma) from the near-cathode region due to thermal conductivity, the heat flux density carried away from the cathode surface due to the energy density of electrons leaving the cathode as a result of secondary electron emission and thermionic emission, and the energy flux density transferred by reverse electrons to the cathode. The second term on the right takes into account the density of the energy flux carried away due to the evaporation of atoms and molecules from the cathode surface:

(14) $$n\cdot Q_{evp}=-L\sum \Gamma_{i}m_{i},$$

where $\Gamma_{i}$ is the flux of atoms and molecules of carbon, *L* is the heat of the vaporization of graphite. The third term in (13) takes into account the density of the energy flux to the cathode due to the deposition of carbon particles on the cathode surface from the discharge zone:

(15) $$n\cdot Q_{dep}=L\sum \Phi_{i}m_{i},$$

where $\Phi_{i}$ is the flux of carbon atoms and molecules from the plasma to the electrode surface.

The boundary condition for Equation (6) on the anode surface from the side of the plasma region (*x* = *L*) was written similarly to (13):

(16) $$n\cdot Q_{a}=n\cdot \left(\sum_{i}^{}Q_{i}+Q_{evp}\right).$$

In this case, the first term on the right takes into account the energy fluxes to the anode due to thermal conductivity, due to the energy transfer by electrons to the anode, and the energy transfer by ions to the anode in the case of a negative anode potential drop. The second term on the right side of (16) is written similarly to (14).

For the continuity of Equation (1), written for particles evaporated from the electrode surface (for atomic and molecular particles of carbon), the boundary condition for the flow must take into account the evaporation of the material. It was assumed that near the electrode surface, there is a Knudsen layer with vapor pressure $p_{\mathrm{sat},C}$. The mass transfer of the electrode material from the solid phase to the gaseous phase is described using the Hertz–Knudsen–Langmuir equation [59,60]:

(17) $$\Gamma_{vap}=\sum_{i=1}^{3}\left(p_{\mathrm{sat},Ci}\left(T\right)-p_{Ci}\right)\sqrt{\frac{M_{Ci}}{2\pi k_{B}T}},$$

where $p_{Ci}=n_{Ci}kT$ are the partial pressures of atomic and molecular (dimers and trimers) carbon particles in the arc plasma for graphite electrodes; $M_{i}$ is the mass of an atom and carbon molecule, and index *i* takes values from 1 to 3 for graphite electrodes. The vapor pressure of atomic and molecular particles of carbon near the electrode surface was determined using the following relations:

(18) $$\log_{10}\left(Gp_{sat,i}\right)=-\frac{A_{i}}{T_{c,a}}+B_{i},$$

where $p_{1}$, $p_{2}$, $p_{3}$ are the partial pressures of C, C_2_, C_3_, vapors, and $T_{C}$ is the temperature of the electrode material. The coefficients $A_{1}=37277.3$, $B_{1}=8.143$, $A_{2}=42332.6$, $B_{2}=9.693$, $A_{3}=40296.0$, $B_{3}=9.811$ for carbon were taken from [61,62].

**Figure 1:**
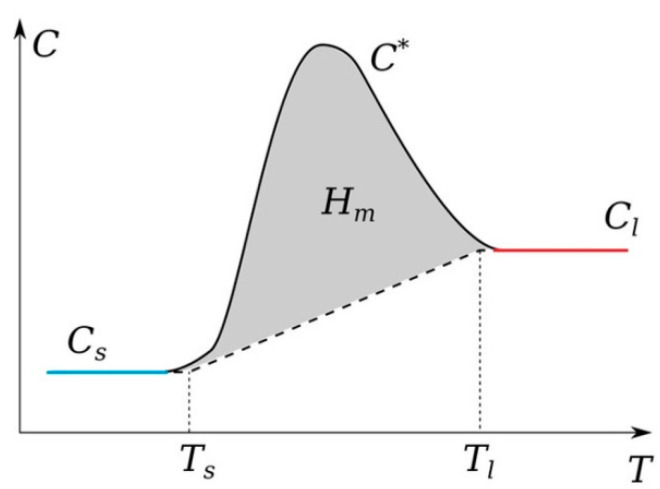
Schematic behavior of the heat capacity for the model account of the phase transition.

It should be noted that graphite is one of the refractory elements. A fairly large number of works have been devoted to the study of its thermophysical parameters, including the melting and evaporation temperatures [63,64]. In addition, it should be noted that the temperature range in which it can exist in the liquid state is small; there are also works in which it is assumed that graphite sublimates [64]. As experiments and preliminary numerical calculations show, in the arc mode, intense heating is observed on the anode surface. It is the anode in the arc discharge mode that experiences a phase transition; we can observe its erosion and a reduction in longitudinal dimensions due to evaporation from the surface into the gas-discharge gap [65]. To account for the phase transition, the apparent heat capacity method was used. According to this method, a phase transition function α(T) is introduced to ensure a smooth transition between the solid and liquid phases in a given interval ΔT near the phase transition temperature $T_{m}$. The heat capacity of the two phases in this interval is expressed as $C_{p}=C_{p,s}(1-\alpha )+C_{p,l}\alpha$ (Figure 1). For a solid phase, it is assumed α=0, and for a liquid, it is assumed α=1. The latent heat of melting $H_{f}$ is included as an additional term in $C_{p}$, i.e.,

(19) $$C_{p}=C_{p,s}(1-\alpha )+C_{p,l}\alpha +H_{f}\frac{d\alpha }{dt}$$

### 2.2. Elementary Processes in Helium Plasma

To describe the elementary processes in a discharge in helium, the works of four teams of researchers were taken as the basis: the works of Donko Z. et al. [66,67] and Bogdanov E.A. et al. [68], who were devoted to various options for the fluid modeling of microdischarges in helium, alongside the work of the scientific group of V. M. Donnelly [69], as well as the work of R. Deloche et al. [70]. We considered three kinds of excited helium atoms: metastable triplet and singlet states, andone effective excited level (with the principal quantum number n = 3), two kinds of positive ions (see Table 1), and one kind of excited (metastable state) of molecular helium. A set of plasma-chemical reactions is presented in Table 2.

### 2.3. Elementary Processes in Argon Plasma

In our previous studies, as well as studies carried out in [48,51,54,55], we showed that in the arc mode, to describe the discharge in argon, we can restrict ourselves to a reduced set of elementary processes. Therefore, in describing the discharge in argon, we used the elementary processes from [54,55]. In addition to electrons, the following states: e, Ar^+^, Ar_2_^+^, Ar*, Ar_2_*.

### 2.4. Kinetics of Elementary Processes Involving Atoms and Molecules of Carbon

Next, we consider a plasma-chemical model with the participation of atomic and molecular particles of carbon entering the gas-discharge gap during the evaporation of graphite electrodes. The considered states of carbon particles are presented in Table 3. The species in the model include atomic, diatomic, and triatomic carbon in ground states (C, C_2_, C_3_), excited states ($C_{}^{*}$, $C_{2}^{*}$, $C_{3}^{*}$), and ionized states ($C_{}^{+}$, $C_{2}^{+}$, $C_{3}^{+}$).

A complete set of plasma-chemical reactions involving atomic and molecular particles of carbon is presented in Table 4. Previously, this set was considered in [52,62]. In addition, electron dissociation [73] was taken into account, as well as Penning ionization of carbon atoms and molecules in a discharge with helium.

## 3. Results and Discussion

**Figure 2:**
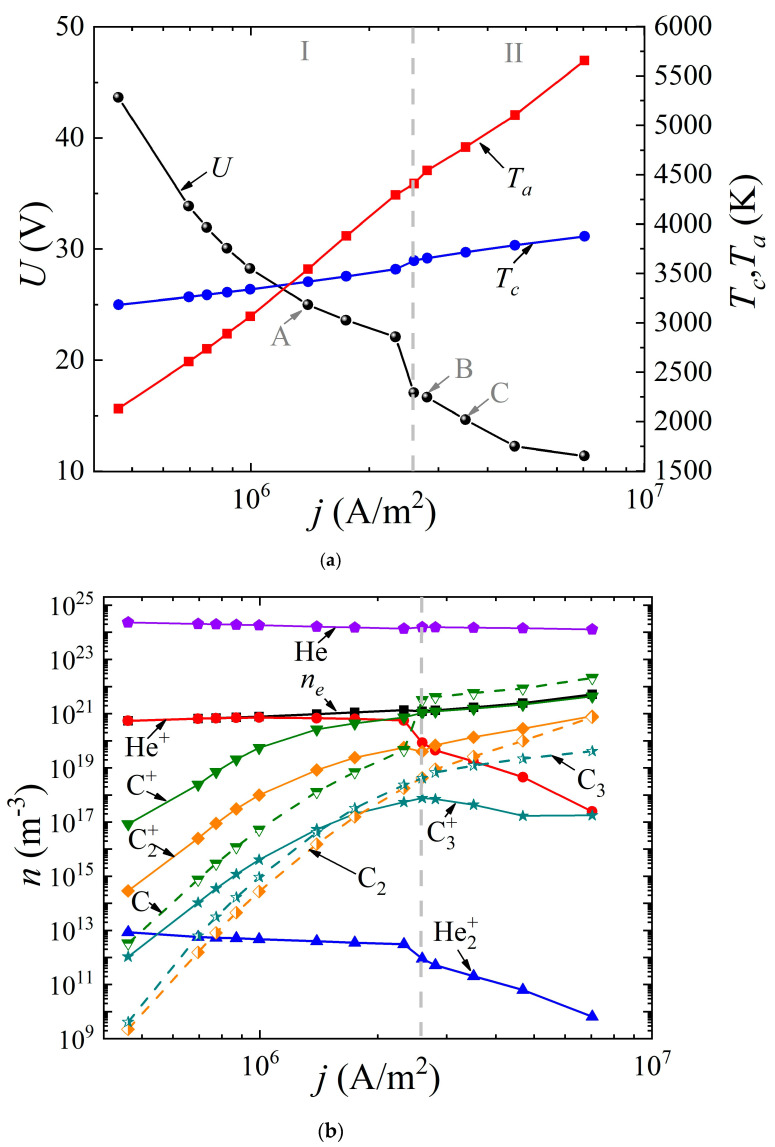
Dependences (**a**) of the voltage across the discharge gap and the temperature of the cathode and anode surfaces on the current density, as well as (**b**) of the concentrations of electrons, ions, and neutral carbon particles averaged over the discharge gap in an arc discharge in helium.

**Figure 3:**
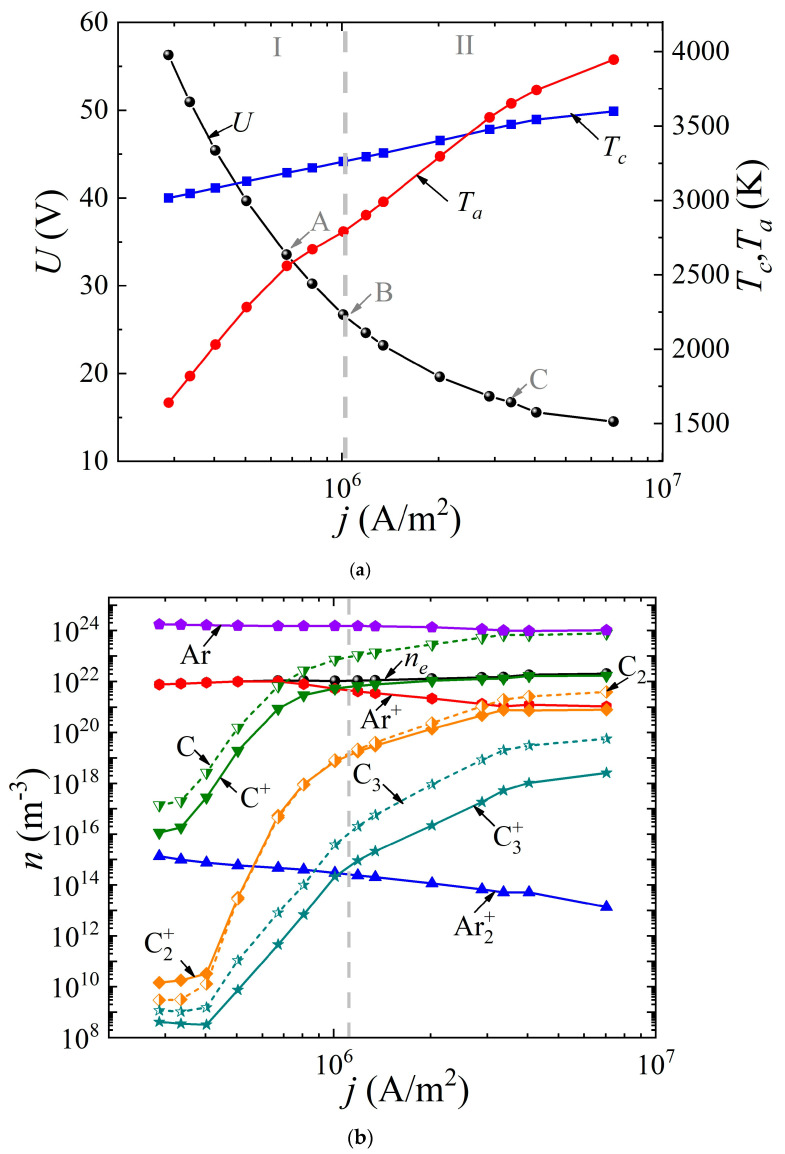
Dependences (**a**) of the voltage across the discharge gap and the temperature of the cathode and anode surfaces on the current density, as well as (**b**) of the concentrations of electrons, ions, and neutral carbon particles averaged over the discharge gap in an arc discharge in argon.

The system of Equations (1)–(8) was solved with the appropriate boundary conditions according to the method presented by us earlier in [49]. It was assumed that the discharge had a uniform structure in the radial direction, so the 1D geometry was considered. The cathode and anode lengths were assumed to be the same and equal to 20 mm; the interelectrode distance varied from 0.4 to 2 mm. The buffer gas pressure (helium or argon) was 760 Torr. The voltage at the source was set equal to 5 kV. By varying the ballast resistance *R_bal_* from 300 Ω to 500 kΩ, the dependences of the discharge voltage drop *U*(*j*) (“CVC”) were obtained, as well as the dependences of the surface temperature of the cathode *T_c_*(*j*) and the anode *T_a_*(*j*) on the discharge current density *j* (Figure 2a and Figure 3a) for the discharge with graphite electrodes in the buffer inert gas helium and argon, respectively. In addition, the average values of the densities of electrons, various types of ions, atoms, and molecules of carbon are presented depending on the current density in the arc in helium (Figure 2b) and argon (Figure 3b), respectively.

As can be seen from Figure 2a and Figure 3a, in both cases, a falling dependence *U(j*) is observed. It can be seen that in helium arc discharge at a current density of 2.5 × 10^6^ A/m^2^, there is a small jump in the potential drop across the discharge gap, equal to ~6 V. Such a jump in the potential drop is often found in the experimental data and is interpreted as a transition to a hissing arc [75]. Apparently, it is associated with intense evaporation of the anode material. But in the argon arc discharge, the characteristic is monotonic. In the helium arc discharge, in the range of current densities up to 1.25 × 10^6^ A/m^2^ and in argon up to 2.5 × 10^6^ A/m^2^, respectively, the cathode surface temperature exceeds the anode surface temperature. As the current density increases, a transition to a new regime occurs, within which the anode surface temperature exceeds the cathode surface temperature and the process of ablation from the anode becomes dominant. This is the so-called anodic arc.

The vertical dashed line corresponding to the current density of 2.5 × 10^6^ A/m^2^ in the helium arc (Figure 2) and 1.1 × 10^6^ A/m^2^ in the argon arc (Figure 3) divides the range of current densities into two modes. Let us analyze these two modes in more detail. To do this, consider Figure 2b and Figure 3b. It can be seen that in the first I mode, in the range of current densities not exceeding 2.5 × 10^6^ A/m^2^ in helium and not exceeding 1.1 × 10^6^ A/m^2^ in argon, respectively, the dominant ion is the atomic buffer gas ion, helium or argon. As the current density increases in the second II mode, the atomic carbon ion becomes the dominant ion. In other words, the effect of changing the plasma-forming ion is observed. This effect is associated with high densities of atomic carbon particles evaporated in the discharge gap, low ionization energies, and high values of the impact ionization cross sections of carbon atoms compared to helium or argon atoms.

In this case, since the values of the ionization potentials of helium (24.6 eV) and carbon (11.26 eV) differ significantly, as well as the values of the maxima of the ionization cross sections, a jump is observed, both in the dependences *U*(*j*) and in the dependences of the average values of the densities of the considered plasma particles (Figure 3a). The close values of the ionization potentials of argon (15.8 eV) and carbon (11.26 eV), as well as the maxima of the ionization cross sections, lead to monotonic dependences *U*(*j*), and plasma particle densities averaged over the discharge gap.

It should be noted that with a further increase in the current density in an arc discharge in helium, the second most important ion becomes the molecular carbon ion C_2_^+^. The role of the molecular ion of the buffer gas He_2_^+^ or Ar_2_^+^ is insignificant, and decreases with increasing current density. The role of the molecular carbon ion C_2_^+^ sharply increases at high current densities in the range of 2 × 10^6^–3 × 10^6^ A/m^2^ and higher.

The following fact is also noteworthy: in the argon arc discharge, in the entire considered range of current densities, the neutral components of carbon C dominate over carbon ions, with the exception of the molecular carbon ion C_2_^+^, whose density is close to the density of the neutral component C_2_. Another feature is observed in helium arc discharge.

In the first I mode, in which the buffer helium ion is the dominant type of ion, the concentration of evaporated carbon ions C^+^, C_2_^+^ exceeds the density of neutral evaporated particles C, C_2_, C_3_. In this case, the main mechanism for the formation of ions of evaporated particles is impact ionization, which is associated with rather high values of the electron temperature or, more precisely, the given value of the self-consistent electric field strength in the discharge gap. In other words, the value of the electric field strength in the discharge is high enough to maintain the discharge (the discharge “burns on helium”), and this field ensures complete ionization of the evaporated carbon particles.

Next, we consider the distribution of densities of different types of particles along the discharge gap of an arc discharge in helium and argon for various current densities.

**Figure 4:**
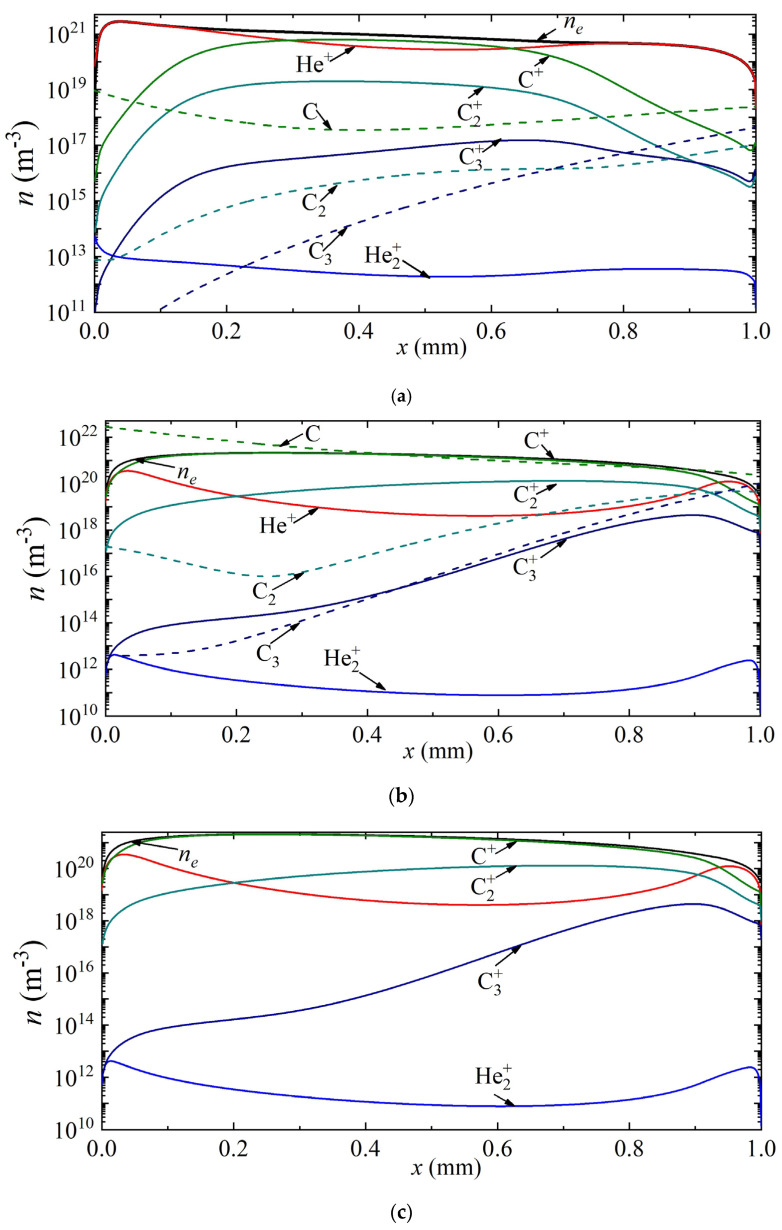
Distribution of the densities of charged and neutral particles in helium arc discharge for different current densities on the CVC corresponding to the points (**a**) A, (**b**) B, and (**c**) C.

Thus, Figure 4 shows the density distributions of electrons, various types of ions, as well as neutral carbon particles along the discharge gap for current densities of 7 × 10^5^ A/m^2^, 2.5 × 10^6^ A/m^2^, 3.5 × 10^6^ A/m^2^, corresponding to points A, B and C in Figure 2a. It can be seen that at a current density of 7 × 10^5^ A/m^2^, the helium ion is the dominant type of ion. The maximum densities of carbon ions C^+^, C_2_^+^, C_3_^+^ are observed in the center of the discharge gap, and reach values of 1.24 × 10^19^ m^−3^, 1.35 × 10^17^ m^−3^, 5.5 × 10^14^ m^−3^, respectively. The density distribution of neutral carbon atoms has a weak minimum at the center of the discharge gap. The maximum values of C_2_ and C_3_ densities are observed near the anode.

With an increase in the current density to a value of 2.5 × 10^6^ A/m^2^, corresponding to point B, the atomic carbon ion becomes the main plasma-forming ion along almost the entire length of the discharge gap; only in a narrow near-cathode region and also in the near-anode region is the dominance of helium ions observed. The density maxima of neutral carbon particles are observed near the electrode surfaces: C near the cathode, and C_2_ and C_3_ near the anode.

With a further increase in the current density to a value of 3.5 × 10^6^ A/m^2^, the dominant ion in the entire discharge gap is the atomic carbon ion. The second most important ion in the cathode region is the atomic argon ion He^+^, and in the rest of the region, the molecular carbon ion C_2_^+^. The character of the distribution of neutral carbon particles does not change.

**Figure 5:**
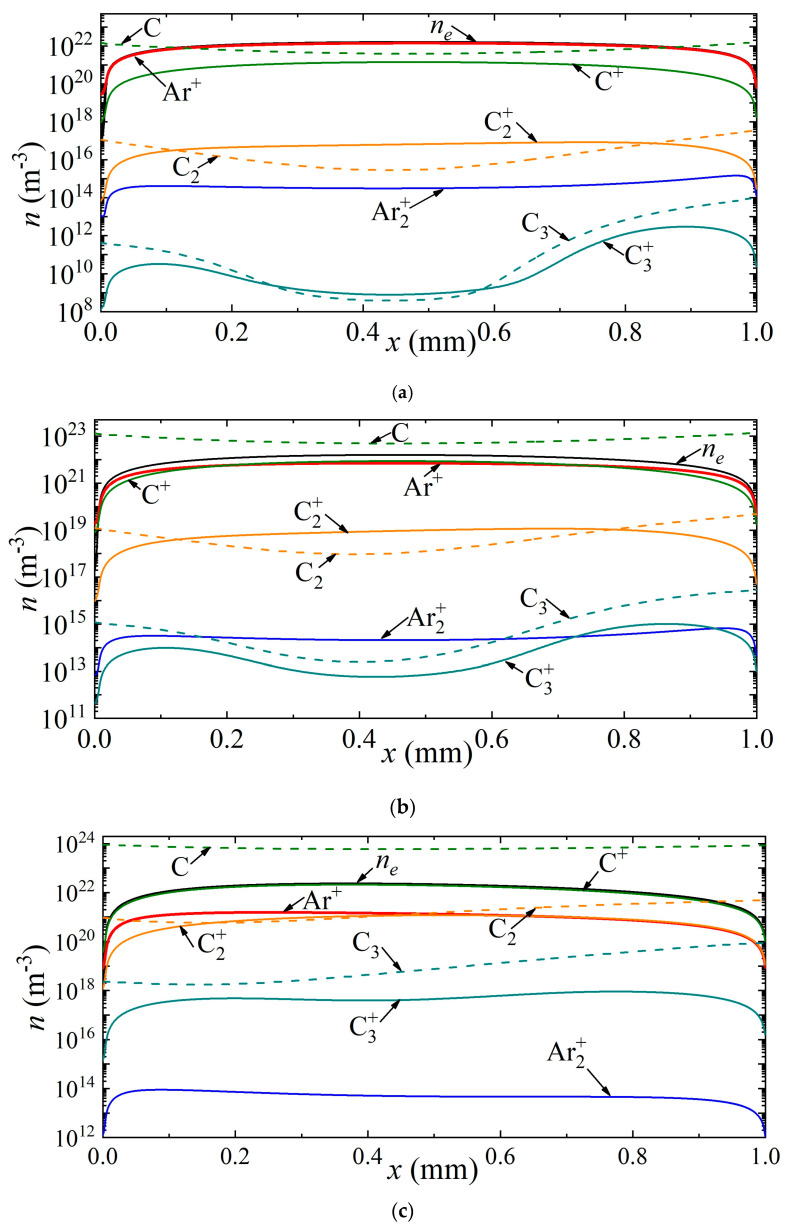
Distribution of the densities of charged and neutral particles in argon arc discharge for different current densities on the CVC corresponding to the points (**a**) A, (**b**) B, and (**c**) C.

Similar distributions are shown in Figure 5 for argon arc discharge. Three points A, B, and C on the CVC (Figure 3a) are considered, corresponding to current densities of 7 × 10^5^ A/m^2^, 1.0 × 10^6^ A/m^2^, and 3.5 × 10^6^ A/m^2^. In the case of the first point A, the dominant ion is the argon ion.

The density distributions of neutral carbon particles have maxima near the electrodes. With an increase in the current density to 1.0 × 10^6^ A/m^2^, the carbon ion becomes the dominant ion in almost the entire discharge gap, except for the near-electrode regions, in which the argon ion predominates. With a further increase in the current density, the carbon ion dominates the entire length of the discharge gap.

**Figure 6:**
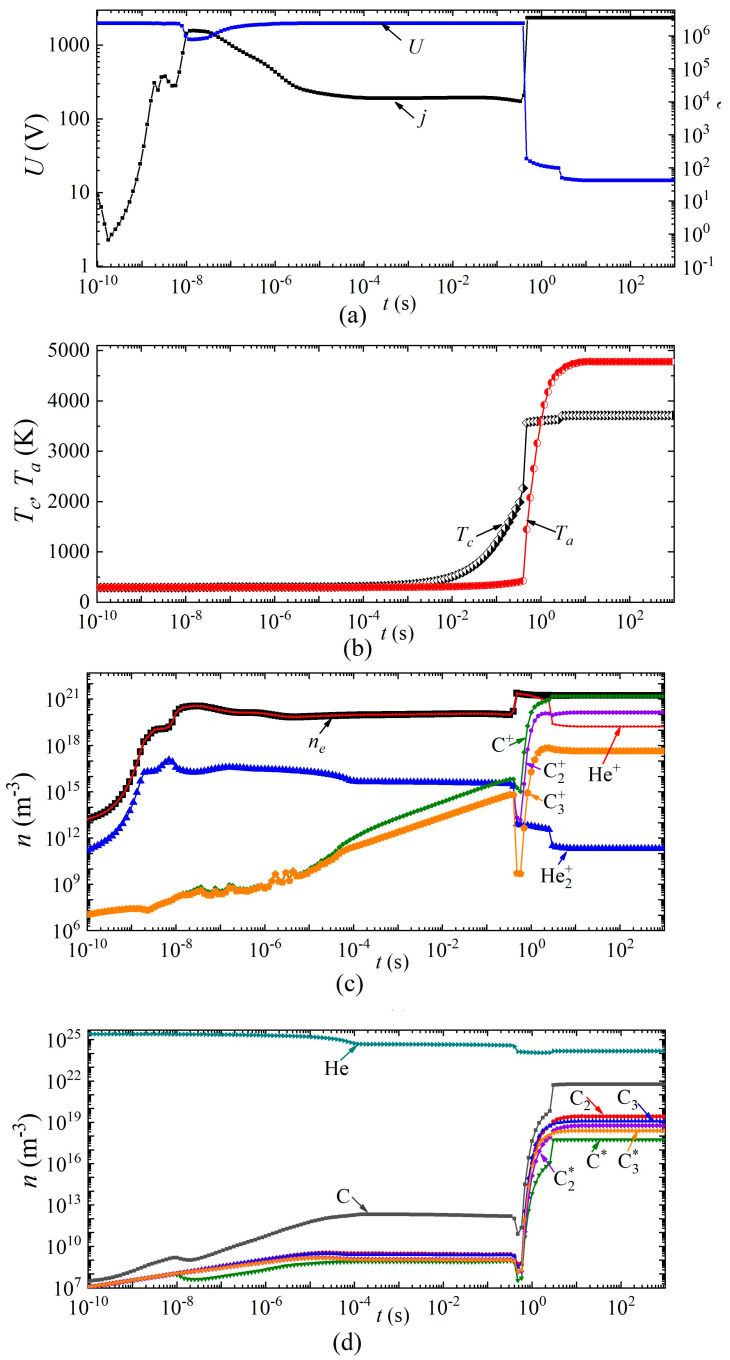
Dynamics of arc discharge parameters in helium: (**a**) current density and voltage, (**b**) cathode and anode surface temperatures; densities of (**c**) charged, (**d**) excited, and neutral particles.

**Figure 7:**
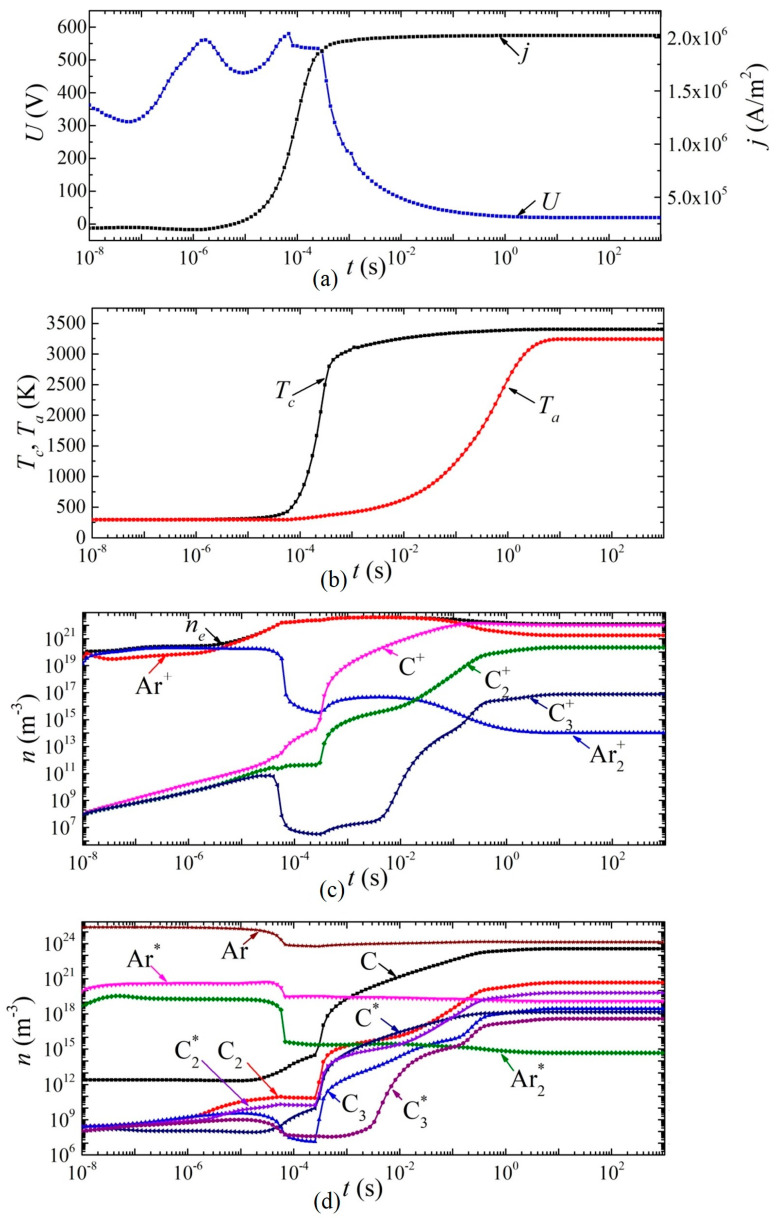
Dynamics of arc discharge parameters in argon: (**a**) current density and voltage, (**b**) cathode and anode surface temperatures; densities of (**c**) charged, (**d**) excited, and neutral particles.

Next, we consider the dynamics of establishing the main parameters of the arc discharge at a current density *j* = 2 × 10^6^ A/m^2^ in helium and argon, respectively (Figure 6 and Figure 7). The breakdown of the discharge occurs at times of the order of 10^−9^ s. At times of the order of several tens of nanoseconds, a glow discharge is established.

Next, the processes associated with the heating of the gas in the discharge gap and a decrease in the concentration of neutral particles are switched on, while the combustion voltage changes, and by the time a change of ~5 × 10^−6^ s has occurred in the discharge in helium (and ~8 × 10^−5^ s in argon), there is a glow discharge with a voltage drop across the discharge gap of ~1900 V for helium and ~500 V for argon, respectively.

In this mode, intense heating of the cathode surface occurs in the time interval from 5 × 10^−6^ s to 0.4 s in helium and from 8 × 10^−5^ s to 3 × 10^−4^ s in argon. Thus, the cathode surface temperature increases from 340 to 2200 K in helium and from 530 K to 2600 K in argon. Further, the discharge begins to switch to the arc mode. Moreover, this transition in a discharge in helium is characterized by two jumps: in the first, the voltage drops from 1900 V to 28–20 V, and in the second, from 20 V to 12 V. The first jump is associated with the transition from a glow discharge to an arc, and the second with a change in the plasma-forming ion. An abrupt character change is also observed during the transition from a glow discharge to an arc, and during a change in the plasma-forming ion in the dynamics of the densities of charged, excited, and neutral particles.

In an arc discharge in argon, such a transition occurs in a smooth (monotonic) manner. From the moment of time ~8 × 10^−5^ s, the concentration of carbon atoms and molecules in the discharge gap, as well as their ions and excited states, begins to increase. Moreover, after 0.2 s, the atomic carbon ion C^+^ becomes the dominant type of ion. After 5 ms, the voltage drop across the arc discharge is less than 100 V, that is, it can be argued that the discharge has switched to the arc mode. In this mode, after ~5 s, a constant current density *j* = 2 × 10^6^ A/m^2^ is established, and there is an increase in the temperature of the cathode surface to 3390 K and the anode surface to 3200 K.

Additional numerical calculations were carried out according to the conditions of the experiments carried out in [76]. For this, it was assumed that the pressure in the interelectrode gap is 500 Torr. The anode diameter was assumed to be 0.65 cm, and the ablation rate was considered after the arc was ignited at a time of 60 s.

**Figure 8:**
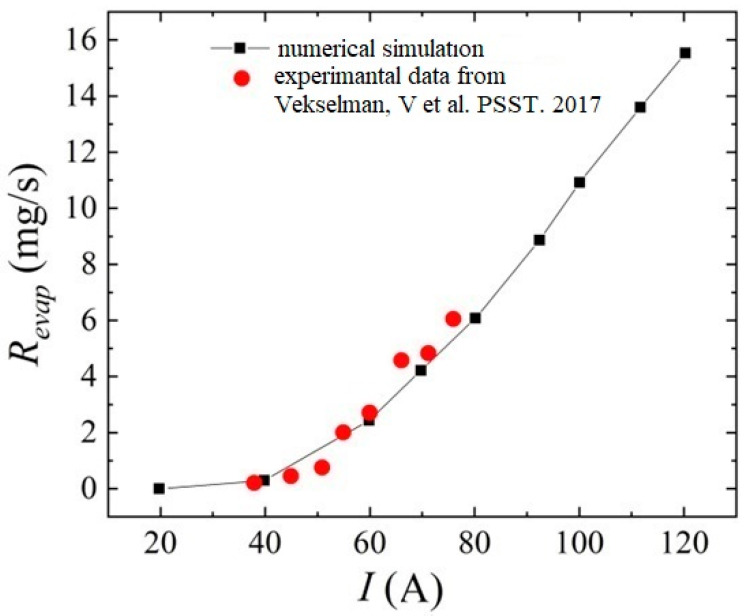
Comparative analysis of the evaporation rate of carbon particles from the anode surface obtained in the framework of modeling and experimental data from the work [76].

Figure 8 presents a comparative analysis of the rate of evaporation of carbon particles from the anode surface from [76] and those obtained in the framework of the formulated model in a helium discharge. We can see a fairly good quantitative agreement between the results, which indicates the reliability of the calculated data obtained.

## 4. Conclusions

Thus, in this work, the arc discharge model, which describes the processes in the discharge gap and electrodes in a unified way [48,49,50,51,52], was further developed, taking into account the processes occurring in the discharge gap and in the electrodes in a unified way. In this case, the process of the evaporation of particles from the anode surface was additionally taken into account. Numerical calculations were considered for arc discharges in helium and argon with graphite electrodes. Additionally, elementary processes involving carbon particles evaporating from the anode surface were taken into account. It is shown that during an arc discharge, the potential jump is observed in the dependence of voltage on current density, which corresponds to a change in the arc discharge regime, in which a change in the plasma-forming ion is observed. In the case of an argon arc, this transition is smooth.

This difference is due to the fact that the ionization potentials, as well as the ionization cross sections, differ significantly for helium and carbon, and are close in magnitude for helium and argon. The density distributions of charged and neutral particles of an inert gas and evaporating gases are presented for different CVC points.

The formulated model and the performed numerical experiments are a convenient tool for the development of modern plasma-chemical reactors based on arc discharges for the synthesis of carbon nanostructures. In particular, on the basis of the formulated model, conditions for the optimal synthesis of nanostructures in terms of pressure and input power can be predicted.

## Figures and Tables

**Table 1:** Considered states of the helium atom in an arc discharge.

№	Symbol	Energy (eV)	Stat. Weight	Effective Level Components
1	He	0	1	$1^{1}S_{0}$
2	He(T)	19.8196	3	$2{\;}^{3}S_{1}$
3	He(S)	20.6157	1	$2{\;}^{1}S_{0}$
4	He*	23.02	36	$3{\;}^{3}S_{0},\;$ $3{\;}^{1}S_{1},\;$ $3{\;}^{3}P_{2}^{0},\;$ $3{\;}^{3}P_{1}^{0},\;$ $3{\;}^{3}P_{0}^{0},\;$ $3{\;}^{3}D_{3}^{},\;$ $3{\;}^{3}D_{2}^{},\;$ $3{\;}^{3}D_{1}^{},\;$ $3{\;}^{1}D_{2}^{},\;$ $3{\;}^{1}P_{1}^{0},\;$
5	He^+^	24.5874	1	He^+^
6	He_2_^+^	22.24	1	He_2_^+^
7	He_2_*	17.97	3	He_2_*

**Table 2:** Set of elementary processes in helium plasma.

R	Reaction	Reaction Constant *k_j_*, m^3^/s, or m^6^/s	Description
1	$e^{–}+He\to e^{–}+He$	$f_{0}(\sigma ,w)$ [67,68,69,70,71,72]	Elastic collision
2	$e^{–}+He\to e^{–}+He(T)$	$f_{0}(\sigma ,w)$ [67,68,69,70,71,72]	Excitation
3	$e^{–}+He\to e^{–}+He(S)$	$f_{0}(\sigma ,w)$ [67,68,69,70,71,72]	Excitation
4	$e^{–}+He\to e^{–}+He^{*}$	$f_{0}(\sigma ,w)$ [67,68,69,70,71,72]	Excitation
5	$e^{–}+He\to 2e^{–}+He^{+}$	$f_{0}(\sigma ,w)$ [67,68,69,70,71,72]	Direct ionization
6	$e^{–}+He(T)\to 2e^{–}+He^{+}$	$f_{0}(\sigma ,w)$ [67,68,69,70,71,72]	Stepwise ionization
7	$e^{–}+He(S)\to 2e^{–}+He^{+}$	$f_{0}(\sigma ,w)$ [67,68,69,70,71,72]	Stepwise ionization
8	$e^{–}+He(T)\to He+e^{–}$	$f_{0}(\sigma ,w)$ [67,68,69,70,71,72]	Superelastic collisions
9	$e^{–}+He(S)\to He+e^{–}$	$f_{0}(\sigma ,w)$ [67,68,69,70,71,72]	Superelastic collisions
10	He(S)+e→He(T)+e	$f_{0}(\sigma ,w)$ [67,68,69,70,71,72]	Mixing level
11	He(S)+He→2He	$8\cdot 10^{-21}$ [67,68]	Deexcitation
12	$He^{*}+He\to He_{2}^{+}+2e^{–}$	$8\cdot 10^{-17}$ [67,68]	Associative ionization
13	$He(T)+2He\to He_{2}^{*}+He$	$8.1\cdot 10^{-48}T\exp\left(-\frac{650}{T}\right)$ [69,70]	Conversion to excimers
14	$\begin{array}{l} He(T)+He(T)\overset{\xi }{\to }He^{+}+He+e^{–}\\ \;\;\;\;\;\;\overset{1-\xi }{\to }He_{2}^{+}+e^{–} \end{array}$	$2.9\cdot 10^{-15}{\left(\frac{T}{300}\right)}^{0.5}$ [69,70]	Penning ionization
15	$\begin{array}{l} He(S)+He(S)\overset{\xi }{\to }He^{+}+He+e^{–}\\ \;\;\;\;\;\;\overset{1-\xi }{\to }He_{2}^{+}+e^{–} \end{array}$	$2.9\cdot 10^{-15}{\left(\frac{T}{300}\right)}^{0.5}$ [69,70]	Penning ionization
16	$\begin{array}{l} He(T)+He(S)\overset{\xi }{\to }He^{+}+He+e^{–}\\ \;\;\;\;\;\;\overset{1-\xi }{\to }He_{2}^{+}+e^{–} \end{array}$	$2.9\cdot 10^{-15}{\left(\frac{T}{300}\right)}^{0.5}$ [69,70]	Penning ionization
17	$\begin{array}{l} He(T)+He_{2}^{*}\overset{\xi }{\to }He^{+}+2He+e^{–}\\ \;\;\;\;\;\;\overset{1-\xi }{\to }He_{2}^{+}+He+e^{–} \end{array}$	$2.9\cdot 10^{-15}{\left(\frac{T}{300}\right)}^{0.5}$ [69,70]	Penning ionization
18	$\begin{array}{l} He(S)+He_{2}^{*}\overset{\xi }{\to }He^{+}+2He+e^{–}\\ \;\;\;\;\;\;\overset{1-\xi }{\to }He_{2}^{+}+He+e^{–} \end{array}$	$2.9\cdot 10^{-15}{\left(\frac{T}{300}\right)}^{0.5}$ [69,70]	Penning ionization
19	$\begin{array}{l} He_{2}^{*}+He_{2}^{*}\overset{\xi }{\to }He^{+}+3He+e^{–}\\ \;\;\;\;\;\overset{1-\xi }{\to }He_{2}^{+}+He+e^{–} \end{array}$	$2.9\cdot 10^{-15}{\left(\frac{T}{300}\right)}^{0.5}$ [69,70]	Penning ionization
20	$He^{+}+2He\to He_{2}^{+}+He$	$1.4\cdot 10^{-43}{\left(T/300\right)}^{-0.6}$ [69,70]	Ion conversion
21	$2e^{–}+He_{}^{+}\to He_{}^{**}+e^{–}$	$6\cdot 10^{-32}{\left(T_{e}/T\right)}^{-4.0\pm 0.5}$ [69,70]	Three body recombination
22	$e^{–}+He_{}^{+}+He\to He_{}^{*}+He$	$1\cdot 10^{-38}{\left(T_{e}/T\right)}^{-2.0}$ [69,70]	Three body recombination
23	$e^{–}+He_{2}^{+}+He\to He_{2}^{*}+He$	$5\cdot 10^{-39}{\left(T_{e}/T\right)}^{-1}$ [69,70]	Three body recombination
24	$e^{–}+He_{2}^{+}\to He_{2}^{*}$	$5\cdot 10^{-15}{\left(T_{e}/T\right)}^{-1}$ [69,70]	Dissociative recombination
25	$2e^{–}+He_{2}^{+}\to He_{}^{*}+He+e^{–}$	$4\cdot 10^{-32}{\left(T_{e}/T\right)}^{-4.0\pm 0.5}$ [69,70]	Dissociative recombination

**Table 3:** Set of considered states of atoms and molecules of carbon in an arc discharge.

№	Symbol	Energy (eV)	Comment
1	C	0	-
2	$C^{*}$	8.864	2*p*3*p*(^3^P)
3	$C^{+}$	11.26	-
4	$C_{2}$	0	-
5	$C_{2}^{*}$	2.394	$C_{2}^{*}\to C_{2}$ (Swan bands)
6	$C_{2}^{+}$	11.79	-
7	$C_{3}^{}$	0	-
8	$C_{3}^{*}$	3.062	$C_{3}^{*}\to C_{3}$ (Swing bands)
9	$C_{3}^{+}$	12.00	-

**Table 4:** Set of elementary processes in arc plasma involving carbon atoms and molecules.

R	Reaction	Reaction Constant *k_j_* *	Description
1	C+e→C+e	$f_{0}(\sigma ,w)$ [72]	Elastic Collision
2	$C_{2}+e\to C_{2}+e$	$f_{0}(\sigma ,w)$ [74]	Elastic Collision
3	$C_{3}+e\to C_{3}+e$	$f_{0}(\sigma ,w)$ [74]	Elastic Collision
4	$C+e\to C^{+}+2e$	$f_{0}(\sigma ,w)$$f_{0}(\sigma ,w)$ [74]	Direct ionization
5	$C+e\to C^{*}+e$	$f_{0}(\sigma ,w)$ [52,62]	Excitation
6	$C^{*}+e\to C+e$	$f_{0}(\sigma ,w)$ [52,62]	Deexcitation
7	$C^{*}+e\to C^{+}+e+e$	$f_{0}(\sigma ,w)$ [52,62]	Stepwise ionization
8	$C_{2}+e\to C_{2}^{+}+2e$	$f_{0}(\sigma ,w)$ [52,62]	Direct ionization
9	$C_{2}+e\to C_{2}^{*}+e$	$f_{0}(\sigma ,w)$ [52,62]	Excitation
10	$C_{2}^{*}+e\to C_{2}+e$	$f_{0}(\sigma ,w)$ [52,62]	Deexcitation
11	$C_{2}^{*}+e\to C_{2}^{+}+2e$	$f_{0}(\sigma ,w)$ [52,62]	Stepwise ionization
12	$C_{3}+e\to C_{3}^{+}+2e$	$f_{0}(\sigma ,w)$ [52,62]	Direct ionization
13	$C_{3}+e\to C_{3}^{*}+e$	$f_{0}(\sigma ,w)$ [52,62]	Excitation
14	$C_{3}^{*}+e\to C_{3}+e$	$f_{0}(\sigma ,w)$ [52,62]	Deexcitation
15	$C_{3}^{*}+e\to C_{3}^{+}+2e$	$f_{0}(\sigma ,w)$ [52,62]	Stepwise ionization
16	$C_{2}+e\to e+2C$	$f_{0}(\sigma ,w)$	Electron dissociation
17	$C_{3}+C\to C_{2}+C_{2}$	$1.7\times 10^{9}T^{1.5}\exp\left(-1.958\times 10^{4}/T\right)$ [52,62]	Chemical reactions between heavy species
18	$C_{2}+C_{2}\to C_{3}+C$	$5\times 10^{11}T^{0.5}\exp\left(-3.02\times 10^{3}/T\right)$ [52,62]	Chemical reactions between heavy species
19	$C_{2}+M\to C+C+M$	$4.5\times 10^{18}T^{-1}\exp\left(-7.093\times 10^{4}/T\right)$ [52,62]	Chemical reactions between heavy species
20	$C+C+M\to C_{2}+M$	$1\times 10^{16}T^{-0.5}$ [52,62]	Chemical reactions between heavy species
21	$C_{3}+M\to C+C_{2}+M$	$1.6\times 10^{16}T\exp\left(-8.748\times 10^{4}/T\right)$ [52,62]	Chemical reactions between heavy species
22	$C+C_{2}+M\to C_{3}+M$	$1\times 10^{16}T^{-0.5}$ [52,62]	Chemical reactions between heavy species
23	$C_{3}+C^{+}\to C_{2}^{+}+C_{2}$	$1.7\times 10^{9}T^{1.5}\exp\left(-1.958\times 10^{4}/T\right)$ [52,62]	Dissociation involving ions
24	$C_{2}^{+}+C_{2}\to C_{3}^{}+C^{+}$	$5\times 10^{11}T^{0.5}\exp\left(-3.02\times 10^{3}/T\right)$ [52,62]	Association involving ions
25	$C_{3}^{+}+C\to C_{2}^{+}+C_{2}$	$4.5\times 10^{18}T^{-1}\exp\left(-7.093\times 10^{4}/T\right)$ [52,62]	Dissociation involving ions
26	$C_{2}^{+}+C_{2}\to C_{3}^{+}+C$	$1\times 10^{16}T^{-0.5}$ [52,62]	Association involving ions
27	$C_{2}^{+}+M\to C_{}^{+}+C+M$	$1.6\times 10^{16}T\exp\left(-8.748\times 10^{4}/T\right)$ [52,62]	Dissociation involving ions
28	$C_{}^{+}+C+M\to C_{2}^{+}+M$	$1\times 10^{16}T^{-0.5}$ [52,62]	Association involving ions
29	$C_{3}^{+}+M\to C_{}^{+}+C_{2}+M$	$1.6\times 10^{16}T\exp\left(-8.748\times 10^{4}/T\right)$ [52,62]	Dissociation involving ions
30	$C_{}^{+}+C_{2}+M\to C_{3}^{+}+M$	$1\times 10^{16}T^{-0.5}$ [52,62]	Association involving ions
31	$C_{3}^{+}+M\to C+C_{2}^{+}+M$	$1.6\times 10^{16}T\exp\left(-8.748\times 10^{4}/T\right)$ [52,62]	Dissociation involving ions
32	$C+C_{2}^{+}+M\to C_{3}^{+}+M$	$1\times 10^{16}T^{-0.5}$ [52,62]	Association involving ions
33	$C^{+}+2e\to C^{*}+e$	$8.75\times 10^{-27}T^{-4.5}$ [52,62]	Three body recombination
34	$C_{2}^{+}+2e\to C_{2}^{*}+e$	$8.75\times 10^{-27}T^{-4.5}$ [52,62]	Three body recombination
35	$C_{3}^{+}+2e\to C_{3}^{*}+e$	$8.75\times 10^{-27}T^{-4.5}$ [52,62]	Three body recombination
36	$C_{2}^{+}+e\to C+C$	$f_{0}(\sigma ,w)$ [52,62]	Dissociative recombination
37	$C_{3}^{+}+e\to C+C$	$f_{0}(\sigma ,w)$ [52,62]	Dissociative recombination
38	$C_{2}^{*}\to C_{2}$	7.14 × 10^6^ [52,62]	Radiation
39	$C_{3}^{*}\to C_{3}$	7.14 × 10^6^ [52,62]	Radiation
40	$He_{}^{*}+C\to C^{+}+He+e$	$2.9\cdot 10^{-15}{\left(T/300\right)}^{0.5}$ [52,62]	Penning ionization
41	$He_{}^{*}+C_{2}\to C_{2}^{+}+He+e$	$2.9\cdot 10^{-15}{\left(T/300\right)}^{0.5}$ [52,62]	Penning ionization
42	$He_{}^{*}+C_{3}\to C_{3}^{+}+He+e$	$2.9\cdot 10^{-15}{\left(T/300\right)}^{0.5}$ [52,62]	Penning ionization

* for reactions 1–16 and 36, 37, the dimension is m^3^/s; for the rest, it is cm^3^/mol/s, or cm^6^/mol^2^/s.

## Data Availability

The datasets used and analyzed in the current study are available from the corresponding author on reasonable request.
